# Negative Refraction with Superior Transmission in Graphene-Hexagonal Boron Nitride (hBN) Multilayer Hyper Crystal

**DOI:** 10.1038/srep25442

**Published:** 2016-05-05

**Authors:** Ayed Al Sayem, Md. Masudur Rahman, M. R. C. Mahdy, Ifat Jahangir, Md. Saifur Rahman

**Affiliations:** 1 Department of EEE, Bangladesh University of Engineering and Technology, Dhaka, Bangladesh; 2 Department of Electrical and Computer Engineering, National University of Singapore, 4 Engineering Drive 3, Singapore; 3 Department of Electrical Engineering, University of South Carolina, Columbia, SC 29208, USA

## Abstract

In this article, we have theoretically investigated the performance of graphene-hexagonal Boron Nitride (hBN) multilayer structure (hyper crystal) to demonstrate all angle negative refraction along with superior transmission. hBN, one of the latest natural hyperbolic materials, can be a very strong contender to form a hyper crystal with graphene due to its excellence as a graphene-compatible substrate. Although bare hBN can exhibit negative refraction, the transmission is generally low due to its high reflectivity. Whereas due to graphene’s 2D nature and metallic characteristics in the frequency range where hBN behaves as a type-I hyperbolic material, we have found graphene-hBN hyper-crystals to exhibit all angle negative refraction with superior transmission. Interestingly, superior transmission from the whole structure can be fully controlled by the tunability of graphene without hampering the negative refraction originated mainly from hBN. We have also presented an effective medium description of the hyper crystal in the low-k limit and validated the proposed theory analytically and with full wave simulations. Along with the current extensive research on hybridization of graphene plasmon polaritons with (hyperbolic) hBN phonon polaritons, this work might have some substantial impact on this field of research and can be very useful in applications such as hyper-lensing.

Negative refraction^1^, hyperbolic metamaterials (HMMs)^2^,^3^,^4^,^5^ and hyper-crystals^6^,^7^ have recently developed a keen interest in the field of photonics and nano-photonics. Hyperbolic metamaterials are relatively old compared to the new concept of hyper-crystals^6^,^7^, which combines the properties of hyperbolic metamaterials and that of photonic crystals, and can find very intriguing photonics applications. Hyper-crystals can be made from the periodic arrangements of metal and HMM, dielectric and HMM or two different HMMs^6^. In HMM, (the core of hyper-crystal) the components of the permittivity tensor have opposite signs in two orthogonal directions, and so the unbounded high-k bulk propagating waves are supported in HMMs^2^,^3^,^4^,^5^. Negative refraction is a very common phenomenon in HMMs and has been demonstrated in visible^8^, mid infrared^9^ and THz ranges^10^ for TM polarized electromagnetic waves. Hyperbolic materials are generally made by using alternate metal dielectric multi-layers^3^,^4^,^5^, metallic nano-rods in a dielectric host^11^,^12^ or they can be hyperbolic naturally such as hBN^13^,^14^,^15^,^16^, which is the latest addition to the hyperbolic (meta) material family^13^,^14^,^15^,^16^. Since a hyper-crystal contains multiple layers of HMMs, natural materials that exhibit HMM behaviors by themselves, are generally the preferred choice^6^,^7^ for constructing hyper crystals.

Graphene, widely used in both photonics and plasmonics in a broad frequency range because of its unique and tunable optical properties, has been recently used in constructing HMMs^10^,^17^,^18^,^19^. hBN is widely used as a substrate material for graphene, as hBN provides an amazing clean environment for graphene having a similar (hexagonal) crystal structure. Hence, hBN insulator is promised to be the most suitable substrate for graphene-based devices^20^,^21^,^22^. Very recently there has been an extensive investigation on the hybridization of graphene plasmon-polaritons with the (hyperbolic) phonon polaritons present in hBN, both theoretically and experimentally^22^,^23^,^24^,^25^,^26^,^27^. But multilayer structure of graphene-hBN stack, which can act as a hyper crystal, has not yet been investigated to the best of our knowledge.

In this article, we have considered the graphene-hBN multilayer structure for constructing a hyper crystal. Graphene shows both better mobility and high relaxation time, if hBN is the substrate material^20^,^21^,^22^. Moreover, hBN has been recently invented as a natural hyperbolic material^13^,^14^,^15^,^16^. In addition, it is possible to fabricate both graphene and hBN in large area using chemical vapor deposition (CVD) techniques^28^,^29^. As a result, the construction of arbitrary heterostructures multilayer stack is feasible. These facts invoke a natural curiosity about the properties and possible applications of graphene-hBN multilayer structure (hyper crystal). In addition, since graphene is an actively tunable material, hyper-crystals made from graphene and hBN can be made tunable by tuning the chemical potential of graphene, giving more degrees of freedom. A theoretical investigation has been done on the properties of graphene-hBN hyper crystals (GhHC) (cf. Fig. 1), especially the one associated with negative refraction. To easily understand the critical role played by graphene in this hyper-crystal, we have also presented an effective medium theory (EMT) based description in the low-k limit. It has been found that GhHC can exhibit an all-angle negative refraction along with a superior transmission. While a bare hBN can also demonstrate negative refraction, transmission turns out to be low due to high reflectivity. Capitalizing on the two-dimensional nature and metallic characteristics of graphene in a particular frequency range, (where hBN shows type-I HMM behavior), it is possible to strongly suppress reflection from the hyper-crystal without any adverse effect on the negative refraction properties. Moreover, graphene being an actively tunable optical material, its conductivity, and as a result its permittivity, can be modified by changing the Fermi level. So the whole device can be made tunable which adds more functionality to the device. We believe this work will be very useful for future investigations on intriguing properties and applications of GhHC; such as imaging^8^, near field radiative heat transfer^30^,^31^, asymmetric transmission device^32^, especially the ones based on negative refraction with superior transmission which may find practical applications, such as hyper-lensing^33^ and imaging in that particular frequency range of interest.

## Theory

The optical conductivity *σ* of graphene depends on frequency (*ω*), chemical potential (*μ*_c_), relaxation time (*τ*) and temperature (*T*). It can be determined using the Kubo formalisms^34^,^35^ which includes both intra-band and inter-band contributions. Graphene is an optically uni-axial anisotropic material because of its 2D nature, whose permittivity tensor can be given by (when graphene lies in the x-y plane),


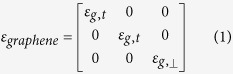


The tangential permittivity of graphene is expressed as,


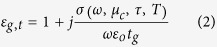


Here *ε*_0_ is the free space permittivity and *t*_*g*_ represents the thickness of monolayer graphene. As graphene is a two dimensional material, the normal electric field cannot excite any current in the graphene sheet. So the normal component of the permittivity is given by *ε*_g,⊥_ = 1^36^. For a few-layer graphene, the tangential permittivity (equation (2)) is the same as that of a mono-layer graphene^37^,^38^. However, for a few-layer graphene, the normal component of permittivity *ε*_g,⊥_ can be approximated as the permittivity (~2) of graphite. In this article, the graphene that we have considered has only a few layers (N = 3, total thickness ~1 nm and the normal component of permittivity *ε*_g,⊥_ = 2). The relaxation time of graphene indicates its purity, and it can vary from fs (femto-second) to ps (pico-second) range. We have considered the relaxation time as *τ* = 200 fs in all calculations. This is a reasonable value as considered in literature for different graphene based devices^10^,^17^,^18^,^19^ along with recent investigation on graphene-hBN system^25^. Considering only the single-Lorentzian form, the principal dielectric tensor components of hBN can be expressed as^15^,^16^,^39^,


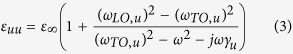


where u = (x, y) represents the transversal (a, b crystal plane) and u = z represents longitudinal (c crystal axis) axes. LO and TO respectively stand for longitudinal and transversal optical phonon frequencies. *ε*_∞_ and *γ* indicate the high-frequency dielectric permittivity and the damping constant, respectively. These data have been taken from^15^. In the frequency range where ω lies between LO and TO frequency, *ε*_*uu*_ becomes negative.

**Figure 1:**
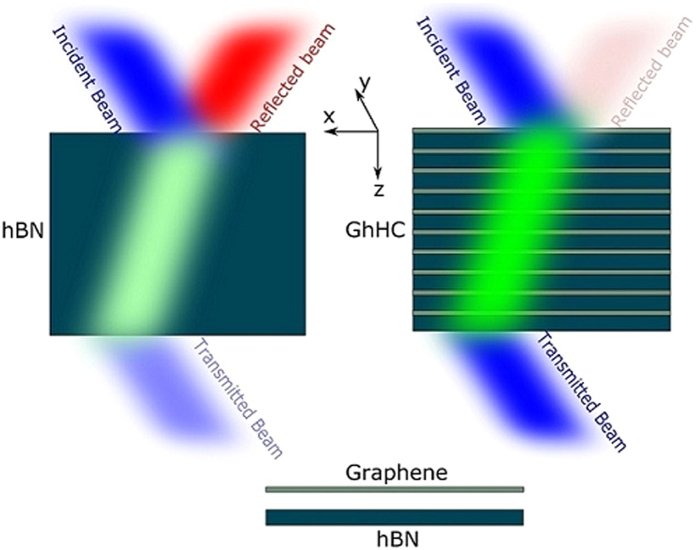
Schematic representation of negative refraction in bare hBN and GhHC. Negative refraction with superior transmission can be achieved in GhHC compared to the bare hBN.

**Figure 2:**
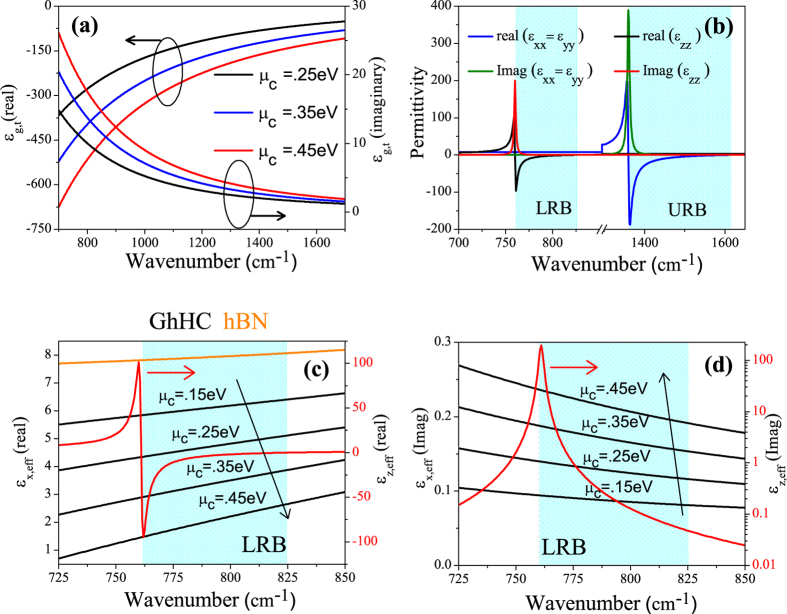
(**a**) Real and imaginary part of the tangential permittivity of graphene as a function of wavenumber for various values of chemical potential of graphene with relaxation time, τ = 200 fs. (**b**) Real and imaginary part of the tangential and perpendicular permittivity of hBN. Shaded regions represent the lower and upper Reststrahlen bands (LRB and URB) (**c**) Real part of the effective tangential (black lines) and perpendicular (red line) permittivity of GhHC for different values of chemical potential of graphene. Real part of the tangential permittivity of hBN (orange line) is also shown for comparison as discussed in the text. (**d**) Imaginary part of the effective tangential (black lines) and perpendicular (red line) permittivity of GhHC for different values of chemical potential of graphene. Thickness of graphene and hBN in the unit cell, d_1_ = 1 nm and d_2_ = 100 nm respectively. Shaded region represents the lower Reststrahlen band (LRB).

Hyper-crystals can be constructed by a stack of^6^: (i) Metal and HMM (ii) Dielectric and HMM (iii) HMM and HMM. Figure 1 shows the schematic of the proposed GhHC. Though this multilayer stack is similar to that of HMMs, this should be considered as hyper crystal. This device is a hyper crystal in this sense that it is a stack of sub-wavelength unit cells containing graphene and hBN (naturally hyperbolic) which falls under category (i). Later we will discuss the difference between commonly known graphene–dielectric multilayer hyperbolic metamaterial^17^,^19^ and GhHC. Figure 2(a) shows the real and imaginary parts of the tangential permittivity of graphene as a function of wave-number for different values of chemical potential. For higher values of chemical potential, the tangential permittivity becomes more negative. In this spectral region, intra-band transition in graphene dominates, and loss is very low due to Pauli blocking of inter-band transition^40^. The chemical potential of graphene can be tuned by either chemical^41^ or electrostatic doping^42^. As shown for the multilayer structure in Fig. 1, a collection of parallel plate capacitors can be made where hBN layers act as insulators, by connecting graphene layers to the positive and negative part of a voltage source sequentially^42^. Then, by varying the voltage between graphene layers, charge concentration on each graphene layer and its chemical potential can be tuned^42^. In the literature, graphene’s chemical potential has been reported to be tuned by up to 1 eV. In our device, much lower values (~0.5 eV) are sufficient for achieving better performance.

Figure 2(b) shows the real and imaginary parts of the calculated tensor components of hBN in the frequency range from 700 to 1650 cm^−1^. In hBN, two Reststrahlen bands are non-overlapping^13^,^15^,^16^ where the term ‘Reststrahlen band’ refers to the frequency range between the transverse (TO) and longitudinal (LO) optical phonons of a polar crystal where at least one of the permittivity tensor component has a real negative part. Due to this non-overlapping Reststrahlen bands, both Type-I and Type-II hyperbolic responses are available in hBN. In the lower Reststrahlen band (LRB), (ω_LO_ = 825 cm^−1^, ω_TO_ = 760 cm^−1^) *ε*_xx_ = *ε*_yy_ > 0 and *ε*_zz_ < 0 and in the upper Reststrahlen band (URB), (ω_LO_ = 1614 cm^−1^, ω_TO_ = 1360 cm^−1^) *ε*_xx_ = *ε*_yy_ < 0 and *ε*_zz_ > 0 and thus leading to the Type I and II hyperbolic response, respectively.

For a general one dimensional anisotropic crystal or hyper-crystal, each layer has the permittivity tensor of the form,


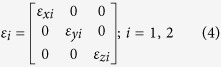


The basic Bloch equation for a p-polarized wave in case of an anisotropic photonic crystal has been derived as,


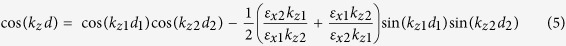


(cf. Supplementary Information for details of the theoretical calculation)

Here d_1_ and d_2_ are the thicknesses of layer-1 and layer-2, respectively, and d = d_1_ + d_2_. k_z1_ and k_z2_ being the propagating wave-number in the first and second layer (respectively), can be given by,


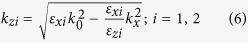


where, *k*_*0*_stands for the wave-number in free-space.

Now, for isotropic case, where both layers have isotropic permittivity (for example, metal-dielectric multilayer), k_z_d ≪ 1 is the sufficient condition for effective medium theory. But for hyper-crystal, the situation is slightly different^6^, because either one or both layers in the unit-cell now can support high-*k* waves (k > k_0_). So, effective medium theory cannot properly describe the physical properties in hyper crystal, such as the band gap, originating from the high-k waves in either or both layers. This failure of effective medium theory in hyper crystal^6^ is different from HMMs^43^. Along with this, we want to mention that, recently this EMT-breaking in HMM has been intelligently used to make an asymmetric transmission device^32^. But due to the difference in EMT-breaking in hyper crystals, such asymmetric transmission device may be difficult to construct using hyper-crystals. But for low-k waves (k < k_0_) and for moderate high-k waves (up to k ~ 20 k_0_), effective medium theory should still work, where layer thicknesses (d_1_ and d_2_) and wavelength (*λ*) play a critical role. Effective permittivity tensor for a hyper-crystal can thus be derived from equation (5) and they are given by,


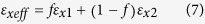



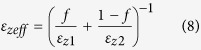


(cf. Supplementary Information for details of the theoretical calculation)

Where, *f* is the filling fraction defined as, 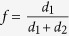


Equations (7) and (8) are the more generalized versions (every layer is anisotropic) of effective medium theory. Figure 2(c) shows the values of the real part of the effective permittivity of GhHC for different values of chemical potential of graphene only for the LRB. Negative refraction for the propagating waves (k < K_0_) occurs only in the lower Reststrahlen band (LRB) where hBN shows type I hyperbolic behavior. In the upper Reststrahlen band (URB), hBN shows type II hyperbolic behavior and propagating waves (in ambient medium, air) (k < K_0_) are not allowed to propagate in either hBN or GhHC. The perpendicular permittivity of graphene is constant because it does not depend on the chemical potential of graphene. As a result, the effective perpendicular permittivity of GhHC is independent of the chemical potential of graphene. On the other hand, the effective tangential permittivity of GhHC is a strong function of the chemical potential of graphene. With higher chemical potential, the effective tangential permittivity of GhHC reduces from bare hBN to a value closer to the permittivity of ambient medium (air). Figure 2(d) shows the values of the imaginary part of the effective permittivity of GhHC for different values of chemical potential of graphene. With higher chemical potential values, the imaginary part of the effective tangential permittivity increases. The imaginary part of the effective perpendicular permittivity of GhHC arises from hBN and has been shown in logarithm scale. The imaginary part of the perpendicular permittivity of GhHC peaks the lower edge of the LRB (ω_TO_ = 760 cm^−1^). But the lower value of the imaginary part of the perpendicular permittivity at the upper edge of the LRB (ω_LO_ = 825 cm^−1^) plays an important role in absorption as discussed later on.

From equations (5), (7) and (8), the wave-vector in the propagating direction for the hyper-crystal (k_z,hc_) can be given by,


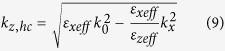


To demonstrate whether effective medium theory works, and to what extent if it does, here we do not put any arbitrary tensor values in equation (4). Instead we consider graphene and hBN as layer-1 and layer-2, as they are the primary topic of interest in this article.

**Figure 3:**
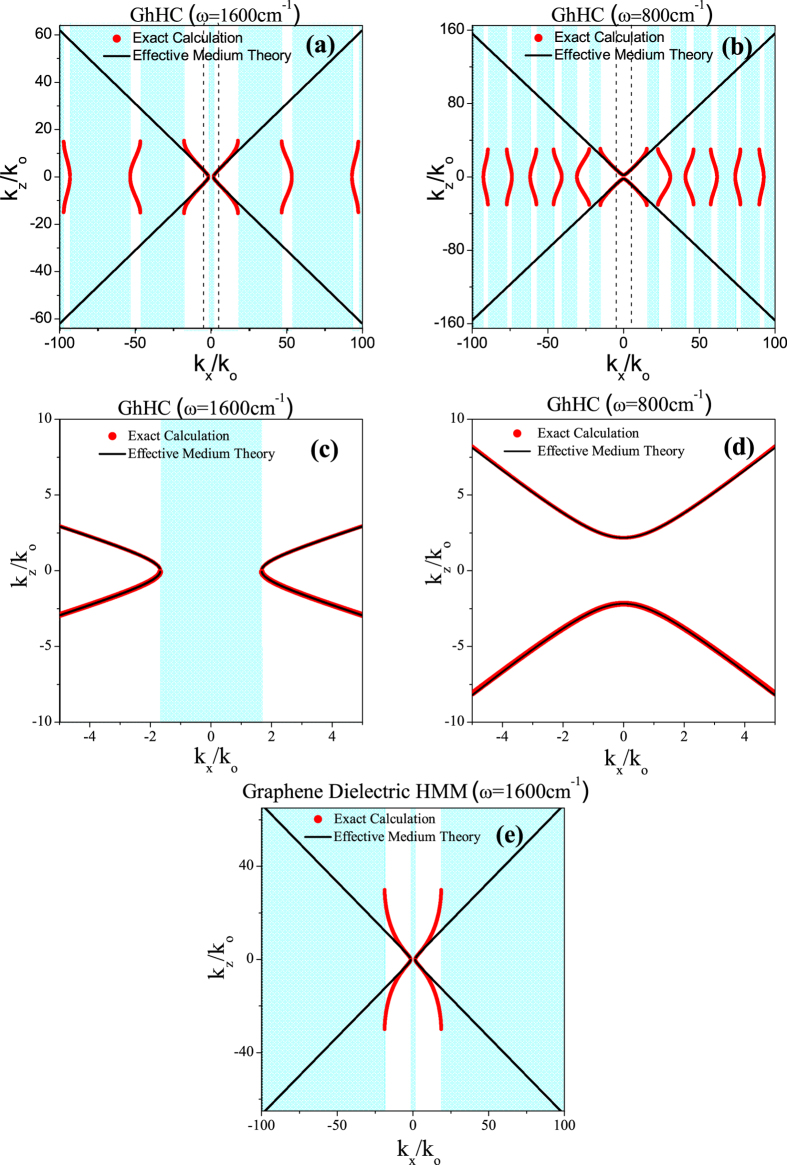
Iso-frequency contour plot of the GhHC (**a**) at wavenumber 1600 cm^−1^ where hBN behaves as a Type-II hyperbolic material (**b**) at wavenumber 800 cm^−1^ where hBN acts as a Type-I hyperbolic material; thickness of graphene and hBN in the unit cell, d_1_ = 1 nm and d_2_ = 100 nm respectively, the chemical potential of graphene, μ_c_ = 0.5 eV. (**c**,**d**) are the zoomed-in view of the specific zone covered by the dashed lines in Fig. 3(**a,b**), respectively. The shaded regions indicate the bandgap in k-space where no propagating wave is allowed. (**e**) Iso-frequency contour plot of a graphene-dielectric multilayer hyperbolic metamaterial; thickness of graphene and dielectric in the unit cell, d_1_ = 1 nm and d_2_ = 100 nm respectively, permittivity of the dielectric ε_d_ = 2.2, the chemical potential of graphene, μ_c_ = 1 eV.

Figure 3(a,b) show the schematic representations of the iso-frequency contour in the wave-vector space for the GhHC (within the loss-less limit) at *ω*_c_ = 1600 cm^−1^ and *ω*_c_ = 800 cm^−1^ respectively for graphene’s chemical potential, μ_c_ = 0.5 eV. Other values of chemical potential of graphene can also be chosen for GhHC. Here, we have chosen μ_c_ = 0.5 eV arbitrarily for illustration purpose. In Fig. 3(e), iso-frequency contour for the graphene-dielectric multilayer (hyperbolic) metamaterial at *ω*_c_ = 1600 cm^−1^ is also shown. The shaded regions indicate the regions where no propagating wave is allowed. The red symbols represent the exact calculation (equation 5) and the black lines represent EMT calculations (equation 9). At *ω*_c_ = 800 cm^−1^ (in the LRB) and *ω*_c_ = 1600 cm^−1^ (in the URB), hBN behaves as a type-I and type-II hyperbolic material, respectively. From Fig. 3(a,b,e) one can clearly distinguish the differences between GhHC and graphene dielectric multilayer (hyperbolic) metamaterial. For GhHC there are multiple bandgaps or forbidden regions where no propagating waves are allowed both in the LRB and URB. Whereas in case of graphene dielectric multilayer (hyperbolic) metamaterial (only Type-II response is available from such graphene dielectric multilayer structure^17^,^19^), after the effective medium breakdown there is complete bandgap in the wave-vector space. Note that the band-gap in the low-k region (cf. Fig. 3(e)) is the characteristic of any type-II hyperbolic metamaterial and higher value of chemical potential of graphene (1 eV) has been used to achieve the desired Type-II hyperbolic behavior. So graphene-hBN multilayer structure must be considered as a hyper crystal^6^. Effective medium theory clearly fails to describe the propagation behavior and the band gap for the high-k waves. Figure 3(c,d) represent the close-up view of iso-frequency contour; the specific zone covered by the dashed lines presented in Fig. 3(a,b), respectively. It can be clearly observed that effective medium theory can adequately represent the propagation behavior at low-k and moderate high-k waves (up to k ~ 20 k_0_).

Any (meta) material which possess hyperbolic dispersion (HMM or hyper crystals), negative refraction especially the all-angle negative refraction will be available from such (meta) materials^2^,^3^,^7^,^8^,^9^,^10^, and this has been demonstrated both theoretically^7^,^8^,^10^ and experimentally^3^,^9^. So from the (hyperbolic) dispersion curves in Fig. 3, it is clearly visible that negative refraction will also be available in GhHC. Here, we consider only the LRB, where hBN behaves as a Type-I hyperbolic medium. Angle of refraction for the power flow can be calculated as in^7^ but, as we can use EMT, we can have a simplified equation. From Maxwell’s equations, refraction angles for the time-average power flow or Pointing vector in a uniaxial anisotropic medium can be given by^8^,


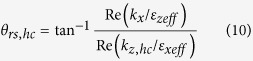


## Simulation and Results

**Figure 4:**
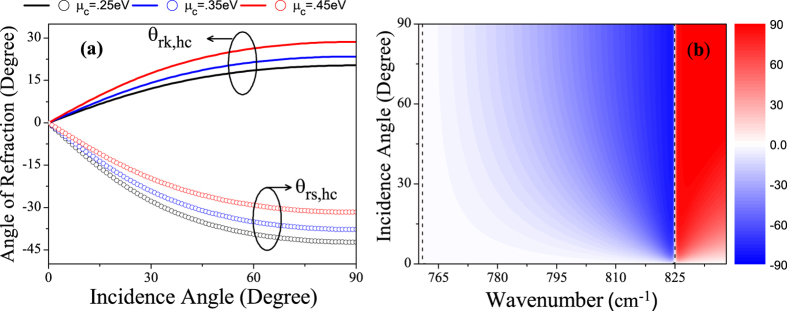
(**a**) Refraction angles for the pointing vector (symbols ‘ο’) and wave-vector (solid lines) for GhHC as a function of incidence angle at wavenumber 800 cm^−1^ for different values of chemical potential of graphene. (**b**) 2D map of the refraction angles for the pointing vector as a function of wavenumber and incidence angle for *μ*_c_ = 0.25 eV. Thickness of graphene and hBN in the unit cell are d_1_ = 1 nm and d_2_ = 100 nm respectively. The black dashed lines indicate the angle of refraction equal to zero.

Figure 4(a) shows the refraction angles of GhHC for the pointing vector (θ_rs,hc_) and wave-vector (θ_rk,hc_) as a function of incidence angle at wavenumber 810 cm^−1^ for different values of chemical potential of graphene. Figure 4(b) shows the 2D map of the refraction angles for the pointing vector (θ_rs,hc_) as a function of wavenumber and incidence angle for *μ*_c_ = 0.25 eV. The black dashed lines indicate the angle of refraction equal to zero (Transition between positive and negative refraction). Only in the LRB (760 cm^−1^ ≤ *ω* ≤ 825 cm^−1^), the effective perpendicular permittivity of GhHC becomes negative (cf. Fig. 2(c)), making negative refraction achievable, which is clearly illustrated in Fig. 4(b). Figure 4(a,b) clearly demonstrate that in the LRB, GhHC exhibit an all-angle negative refraction.

**Figure 5:**
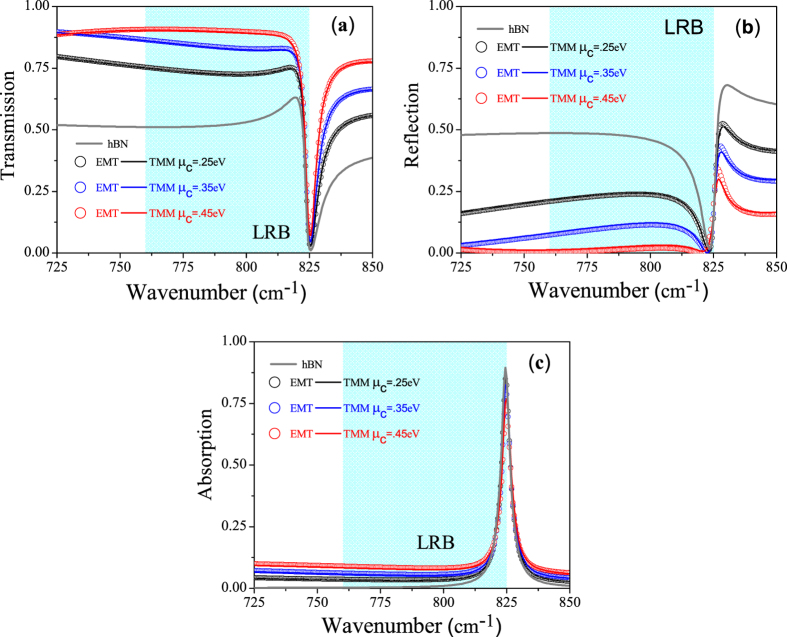
(**a**) Transmission (**b**) reflection and (**c**) absorption coefficient of hBN and GhHC as a function of wavenumber with incidence angle 30°. The solid lines and symbols represent the calculated values for GhHC by the transfer matrix method (TMM) and effective medium theory, respectively, for different values of chemical potential of graphene (0.25 eV, 0.35 eV, 0.45 eV). The solid gray lines represent the calculated values of bare hBN. Total thickness of both bare hBN and GhHC d = ~1 μm, thickness of graphene and hBN in the unit cell, d_1_ = 1 nm and d_2_ = 100 nm respectively, total number of unit cell, N = 10 and the relaxation time of graphene, τ = 200 fs. Shaded region represents the lower Reststrahlen band (LRB).

Figure 5(a–c) show the calculated transmission, reflection and absorption coefficient as a function of wavenumber for GhHC with different values of chemical potential of graphene along with bare hBN (Bare hBN and GhHC have almost the same total thickness). The solid lines and symbols represent the calculated values for GhHC by the general transfer matrix method (TMM)^44^,^45^,^46^ and effective medium theory, respectively. The solid gray lines represent the calculated values of bare hBN. For GhHC, total number of unit cell, N = 10 and the thickness of graphene and hBN in the unit cell, d_1_ = 1 nm and d_2_ = 100 nm respectively with total thickness of the structure equals to ~1 μm. The first interface in the simulation was graphene. (cf. Supplementary Information for the effect of interface (top) layer). For bare hBN in the LRB, in spite of low absorption, transmission is relatively moderate due to high reflection arising from the impedance mismatch (high in plane permittivity of hBN (cf. Fig. 2(c)). But in case of GhHC, transmission from the structure is a strong function of the chemical potential of graphene. Reflection from GhHC can be highly suppressed tuning the chemical potential of graphene. The physics behind higher transmission can be easily understood. Reflection of bare hBN in the type-I hyperbolic region arises due to the higher dielectric tangential permittivity (cf. Fig. 2(c)). But in GhHC, the tangential permittivity is the effective permittivity of graphene and hBN. Because of the high negative permittivity of graphene (cf. Fig. 2(a)), effective tangential permittivity decreases from that of the bare hBN and becomes comparable to the ambient (air) permittivity (cf. Fig. 2(c)). As a result, momentum mismatch or impedance mismatch reduces, and so reflection gets highly suppressed. It has been mentioned earlier that the perpendicular permittivity graphene is small (~2) and also not dispersive. Therefore, it has a little effect on the perpendicular permittivity of the GhHC, which is required for negative refraction (cf. Equation (10)); *ε*_xeff_ being positive, one needs *ε*_zeff_ < 0 to get a negative *θ*_rs,hc_).

Along with transmission, we want to clearly mention about the absorption properties of GhHC. Although the resonance of absorption of both the hBN and GhHC is at the beginning of the lower Reststrahlen band (LRB) (cf. Fig. 2(b,d)), the maximum of absorption is at the end of the band for both bare hBN and GhHC. This is due to the epsilon near zero behavior of the perpendicular permittivity of hBN or GhHC. In the LRB, there are two frequency regions where the normal permittivity of hBN or GhHC becomes zero; one at beginning of the LRB where loss is also high and other at the end of the LRB where loss is relatively low (cf. Fig. 2(d)). It has been already reported in literature that for oblique incidence upon an uniaxial (meta)material at the epsilon near zero condition (for the perpendicular component of the permittivity), high absorption takes place only for small loss or small imaginary part of the perpendicular permittivity^9^,^15^. In our study of GhHC, we have also observed very high absorption at upper edge of the LRB where normal component of the permittivity becomes zero and loss is also small. These observations are in full agreement with^9^,^15^.

**Figure 6:**
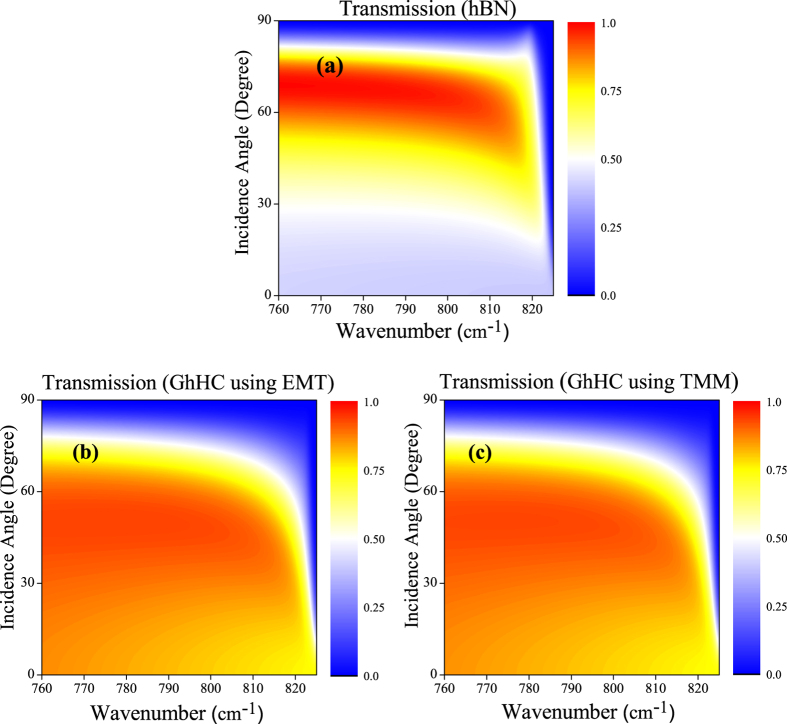
2D map of the transmission coefficient of (**a**) hBN, (**b**) GhHC by effective medium theory and (**c**) GhHC by transfer matrix method as a function of wavenumber and incidence angle. Total thickness of both hBN and GhHC d = ~1 μm. In case of GhHC, thickness of graphene and hBN in the unit cell are d_1_ = 1 nm and d_2_ = 100 nm respectively, total number of unit cell, N = 10, chemical potential of graphene, μ_c_ = 0.4 eV and relaxation time, τ = 200 fs.

Figure 6(a) shows the 2D map of the transmission coefficient as a function of wave-number and incidence angle (up to 90 degree) for bare hBN, whereas Fig. 6(b,c) show the same for GhHC calculated by EMT and TMM^44^,^45^,^46^, respectively. We can clearly observe the complete validation of the effective medium approximation for the low-k waves. Also the superior transmission for GhHC over bare hBN for almost all angles and in the whole LRB (where negative refraction is possible) is clearly observed.

**Figure 7:**
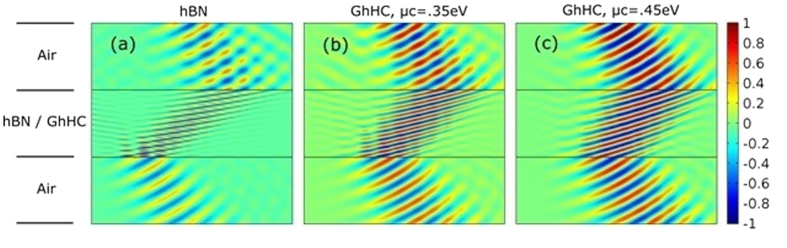
Full wave numerical (Comsol Multi-physics) calculations (Real part of the transverse magnetic field component, H_y_) demonstrating negative refraction of a monochromatic (ω = 810 cm^−1^) TM-polarized Gaussian beam incident from air with incidence angle 30° to (**a**) hBN, and to GhHC for (**b**) *μ*_c_ = 0.35 eV (**c**) *μ*_c_ = 0.45 eV.

Figure 7 shows the real part of transverse magnetic field profile (H_y_) of a p-polarized wave, incident from air to hBN and GhHC (calculated using full wave simulation, Comsol Multiphysics 4.3b) using EMT. Figure 7(a) is for hBN and Fig. 7(b,c) are for GhHC with different chemical potential values of graphene. As described earlier, with higher chemical potential, the tangential permittivity of graphene becomes more negative and tangential permittivity of the GhHC gets closer to that of the ambient medium (air). As a result, lower reflection and higher transmission are achieved without hampering negative refraction. This is clearly illustrated from the full wave numerical simulation in Fig. 7(a–c).

**Figure 8:**
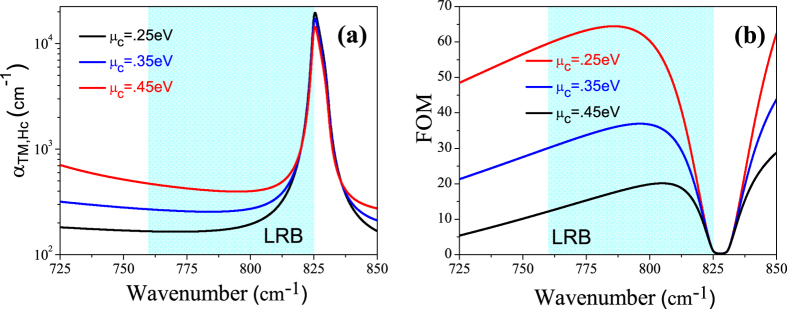
(**a**) The absorption coefficient α_TM,hc_ and (**b**) the figure of merit, FOM, for the GhHC for TM polarization as function of wavenumber for different values of chemical potential of graphene. Shaded region represents the lower Reststrahlen band (LRB).

The FOM = Re(k_z,hc_)/Im(k_z,hc_) is a well-known and accepted value for characterizing materials which possess negative refraction^9^,^47^. Figure 8 shows the absorption coefficient, α_TM,hc_(Im(k_z,hc_)) for TM polarization and the figure of merit (FOM) respectively calculated using the effective medium approximation as a function of wavenumber for different potential values of chemical of graphene. Although for higher chemical potential values, the FOM decreases. But higher transmission is achieved for higher chemical potential values of graphene which mainly occurs due to the reduction of reflection at the ambient medium (air)-GhHC interface.

In conclusion, in this article, we have investigated the properties of graphene-hBN based multilayer structure (hyper crystal). We have found that an all-angle negative refraction is achievable in such a hyper crystal with much higher transmission compared to bare hBN. Transmission and negative refraction properties can be controlled by tuning the chemical potential of graphene. Here negative refraction originates from the optical properties of hBN and transmission from the structure can be fully controlled by graphene without hampering negative refraction. Such properties are quite remarkable, and experimental demonstration of such phenomena should be possible with current experimental setup. We have also presented an effective medium description of the hyper crystal in the low-k limit, which might be useful in theoretical calculations and it has been validated by numerical calculations. We believe that, with the current ongoing extensive investigation on the hybridization of graphene plasmon polaritons with (hyperbolic) hBN phonon polaritons^22^,^23^,^24^,^25^,^26^,^27^, this work could pave the way to the discovery of many intriguing physical phenomena of graphene-hBN multilayer structure (hyper crystal) and enable many interesting applications to emerge within a very short time.

## Additional Information

**How to cite this article**: Sayem, A. A. *et al*. Negative Refraction with Superior Transmission in Graphene-Hexagonal Boron Nitride (hBN) Multilayer Hyper Crystal. *Sci. Rep*. **6**, 25442; doi: 10.1038/srep25442 (2016).

## Supplementary Material

Supplementary Information
