# Supplementary material for: Negative Refraction with Superior Transmission in Graphene-Hexagonal Boron Nitride (hBN) Multilayer Hyper Crystal
Source: Sci Rep. 2016 May 5;6:25442. doi: 10.1038/srep25442 (PMC4857181; doi:10.1038/srep25442)
Supplement: Supplementary Information [file srep25442-s1.pdf]

Supplementary Information for **"Negative Refraction with Superior Transmission in Graphene-Hexagonal Boron Nitride (hBN) Multilayer Hyper Crystal"**

Ayed Al Sayem[1*], Md. Masudur Rahman[1,] M.R.C. Mahdy[1, 2*], Ifat Jahangir[1, 3] and Md. Saifur Rahman[1]

[1]*Department of EEE, Bangladesh University of Engineering and Technology, Dhaka Bangladesh*

[2]*Department of Electrical and Computer Engineering, National University of Singapore, 4 Engineering Drive 3, Singapore*

[3]*Department of Electrical Engineering, University of South Carolina, Columbia, SC 29208, USA*

[*]*ayedalsayem143@gmail.com*

[*]A0107276@nus.edu.sg

Derivation of Bloch Equation and Effective Medium Theory for Anisotropic Photonic Crystal

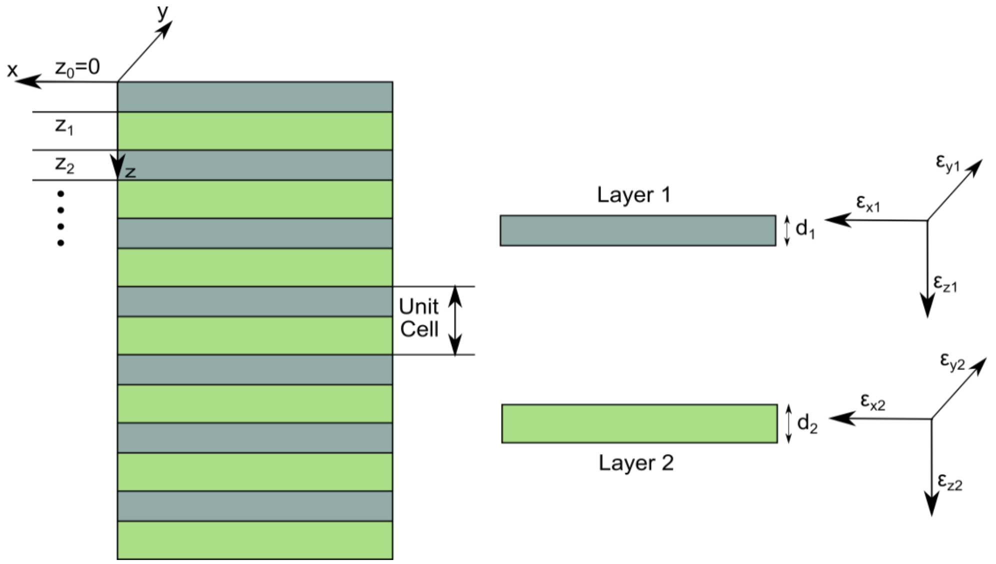

Figure. S1: Schematic figure of the stack of the anisotropic multilayer considered in this study. Two different uniaxial anisotropic materials (layers) with different permittivity tensor

components are stacked in the vertical z direction, the structure is azimuthally (φ) invariant in the x-y plane.

Figure S1 shows the schematic of the proposed structure under study. Permittivity tensor of layer 1 in unit cell can be given by,

$$\boldsymbol{\varepsilon_1} = \begin{bmatrix} \varepsilon_{x_1} & 0 & 0 \\ 0 & \varepsilon_{y_1} & 0 \\ 0 & 0 & \varepsilon_{z_1} \end{bmatrix} \tag{S1}$$

Permittivity tensor of layer 2 in unit cell can be given by,

$$\boldsymbol{\varepsilon_2} = \begin{bmatrix} \varepsilon_{x_2} & 0 & 0 \\ 0 & \varepsilon_{y_2} & 0 \\ 0 & 0 & \varepsilon_{z_2} \end{bmatrix} \tag{S2}$$

For uniaxial anisotropic material, $\varepsilon_{x_1} = \varepsilon_{y_1}$ and $\varepsilon_{x_2} = \varepsilon_{y_2}$

Dispersion relation for the layer 1 and layer 2 in the unit cell can be given by (for $k_y = 0$),

$$\frac{k_{z1}^2}{\varepsilon_{x1}} + \frac{k_x^2}{\varepsilon_{z1}} = k_o^2 \tag{S3}$$

$$\frac{k_{z2}^2}{\varepsilon_{x2}} + \frac{k_x^2}{\varepsilon_{z2}} = k_o^2 \tag{S4}$$

Where, $k_o = \frac{2\pi}{\lambda}$ is the free space wave-vector, λ is the wavelength, k_x and k_z are the x and z component of the wave vector for the anisotropic layers respectively. k_x has to be conserved at each interface. For 2d analysis, we have taken y component of the wave vector, $k_y = 0$ and also the structure is azimuthally (φ) invariant.

For TM (p) polarization, at the *i* th layer, magnetic field component H_y can be expressed as,

$$H_{yi}(x, z) = A_i\, e^{+jk_{zi}(z-z_i)} + B_i e^{-jk_{zi}(z-z_i)};\ z_i < z < z_{i+1} \tag{S5}$$

Where *j* denotes the imaginary part, k_{zi} is the z propagating wave-vector in the *i* th layer.

Here $z_{i+1} - z_i = d_1$ when i=0, 2, 4, … and $z_{i+1} - z_i = d_2$ when i=1, 3, 5, …

From Maxwells equations, tangential components of the electric and magnetic fields at the i th layer can be expressed as,

$$\begin{bmatrix} H_{yi} \\ E_{xi} \end{bmatrix} = M_i \begin{bmatrix} A_i \\ B_i \end{bmatrix} \tag{S6}$$

$$\text{Where } M_i = \begin{bmatrix} e^{-jk_{zi}(z-z_i)} & e^{+jk_{zi}(z-z_i)} \\ \frac{k_{zi}e^{-jk_{zi}(z-z_i)}}{\omega\varepsilon_{xi}} & \frac{-k_{zi}e^{+jk_{zi}(z-z_i)}}{\omega\varepsilon_{xi}} \end{bmatrix} \tag{S7}$$

Where, ω is the angular frequency.

General boundary condition at each interface can be given by,

$$\begin{bmatrix} H_{y(i+1)} \\ E_{x(i+1)} \end{bmatrix}_{z=z_{i+1}} = \begin{bmatrix} H_{yi} \\ E_{xi} \end{bmatrix}_{z=z_{i+1}} \tag{S8}$$

$$\begin{bmatrix} A_{i+1} \\ B_{i+1} \end{bmatrix} = T_{i,i+1}(z_{i+1}) \begin{bmatrix} A_i \\ B_i \end{bmatrix} \tag{S9}$$

$$\text{Where } T_{i,i+1}(z_{i+1}) = [(M_{i+1})^{-1} M_i]_{z=z_{i+1}} \tag{S10}$$

Where,

$$M_{i+1} = \begin{bmatrix} e^{-jk_{z(i+1)}(z-z_{i+1})} & e^{+jk_{z(i+1)}(z-z_{i+1})} \\ \frac{k_{z(i+1)}e^{-jk_{z(i+1)}(z-z_{i+1})}}{\omega\varepsilon_{x(i+1)}} & \frac{-k_{z(i+1)}e^{+jk_{z(i+1)}(z-z_{i+1})}}{\omega\varepsilon_{x(i+1)}} \end{bmatrix} \tag{S11}$$

Now for a periodic photonic crystal (cf. Fig. S1) made from a stack of periodic unit cells containing two anisotropic layers. We label them as layer 1 with thickness d_1 and layer 2 with thickness d_2.

So for i=1 we get,

$$T_{1,2}(z_2) = [(M_2)^{-1} M_1]_{z=z_2}$$

$$T_{1,2}(z_2) = T_{1,2}(d_1) = \frac{1}{2}\begin{bmatrix} e^{-jk_{z1}d_1} + \frac{\varepsilon_{x_2}}{k_{z2}}\frac{k_{z1}}{\varepsilon_{x_1}}\, e^{-jk_{z1}d_1} & e^{+jk_{z1}d_1} - \frac{\varepsilon_{x_2}}{k_{z2}}\frac{k_{z1}}{\varepsilon_{x_1}} e^{+jk_{z1}d_1} \\ e^{-jk_{z1}d_1} - \frac{\varepsilon_{x_2}}{k_{z2}}\frac{k_{z1}}{\varepsilon_{x_1}} e^{-jk_{z1}d_1} & e^{+jk_{z1}d_1} + \frac{\varepsilon_{x_2}}{k_{z2}}\frac{k_{z1}}{\varepsilon_{x_1}} e^{+jk_{z1}d_1} \end{bmatrix} \quad \text{(S12)}$$

Similarly for $(i+1)$ or 2nd layer to $(i+2)$ (3rd) or 1st layer one can find,

$$T_{2,3}(z_3) = T_{2,1}(d_2) = \frac{1}{2}\begin{bmatrix} e^{-jk_{z2}d_2} + \frac{\varepsilon_{x_1}}{k_{z1}}\frac{k_{z2}}{\varepsilon_{x_2}} e^{-jk_{z2}d_2} & e^{+jk_{z2}d_2} - \frac{\varepsilon_{x_1}}{k_{z1}}\frac{k_{z2}}{\varepsilon_{x_2}} e^{+jk_{z2}d_2} \\ e^{-jk_{z2}d_2} - \frac{\varepsilon_{x_1}}{k_{z1}}\frac{k_{z2}}{\varepsilon_{x_2}} e^{-jk_{z2}d_2} & e^{+jk_{z2}d_2} + \frac{\varepsilon_{x_1}}{k_{z1}}\frac{k_{z2}}{\varepsilon_{x_2}} e^{+jk_{z2}d_2} \end{bmatrix} \quad \text{(S13)}$$

Now, Total Transfer Matrix,

$$T = T_{2,1}(d_2)T_{1,2}(d_1)$$

$$= \begin{bmatrix} e^{-jk_{z1}d_1}\left[\cos(k_{z2}d_2) - \frac{j}{2}\left(\frac{\varepsilon_{x_1}}{k_{z1}}\frac{k_{z2}}{\varepsilon_{x_2}} + \frac{\varepsilon_{x_2}}{k_{z2}}\frac{k_{z1}}{\varepsilon_{x_1}}\right) sin(k_{z2}d_2)\right] & e^{+jk_{z1}d_1}\left[-\frac{j}{2}\left(\frac{\varepsilon_{x_1}}{k_{z1}}\frac{k_{z2}}{\varepsilon_{x_2}} - \frac{\varepsilon_{x_2}}{k_{z2}}\frac{k_{z1}}{\varepsilon_{x_1}}\right) sin(k_{z2}d_2)\right] \\ e^{-jk_{z1}d_1}\left[\frac{j}{2}\left(\frac{\varepsilon_{x_1}}{k_{z1}}\frac{k_{z2}}{\varepsilon_{x_2}} - \frac{\varepsilon_{x_2}}{k_{z2}}\frac{k_{z1}}{\varepsilon_{x_1}}\right) sin(k_{z2}d_2)\right] & e^{+jk_{z1}d_1}\left[\cos(k_{z2}d_2) + \frac{j}{2}\left(\frac{\varepsilon_{x_1}}{k_{z1}}\frac{k_{z2}}{\varepsilon_{x_2}} + \frac{\varepsilon_{x_2}}{k_{z2}}\frac{k_{z1}}{\varepsilon_{x_1}}\right) sin(k_{z2}d_2)\right] \end{bmatrix}$$

Let, $\gamma = \frac{\varepsilon_{x_1}}{k_{z1}}\frac{k_{z2}}{\varepsilon_{x_2}}$

$$T = \begin{bmatrix} e^{-jk_{z1}d_1}\left[\cos(k_{z2}d_2) - \frac{j}{2}\left(\gamma + \frac{1}{\gamma}\right) sin(k_{z2}d_2)\right] & -e^{+jk_{z1}d_1}\left[\frac{j}{2}\left(\gamma - \frac{1}{\gamma}\right) sin(k_{z2}d_2)\right] \\ e^{-jk_{z1}d_1}\left[\frac{j}{2}\left(\gamma - \frac{1}{\gamma}\right) sin(k_{z2}d_2)\right] & e^{+jk_{z1}d_1}\left[\cos(k_{z2}d_2) + \frac{j}{2}\left(\gamma + \frac{1}{\gamma}\right) sin(k_{z2}d_2)\right] \end{bmatrix} \quad \text{(S14)}$$

Now, for a periodic lattice,

$$H(z,k) = U_k(z)e^{ik_z(\omega)z} \quad \text{(S15)}$$

$$U_k(z) = U_k(z+d) \quad \text{(S16)}$$

Where, $d = d_1 + d_2$

Where, $k_z(\omega)$ is the bloch wavenumber. Now,

$$k_z(\omega) = \frac{1}{d}\cos^{-1}\left(\frac{1}{2}Trace(T)\right) \quad \text{(S17)}$$

From equations (S14) and (S17),

$$\cos(k_z(\omega)d) = \cos(k_{z2}d_2)\cos(k_{z1}d_1) - \frac{1}{2}\left(\gamma + \frac{1}{\gamma}\right) sin(k_{z2}d_2) sin(k_{z1}d_1) \tag{S18}$$

Now expanding equation (S18) in Taylor series and neglecting the higher order (3rd, 4th,…. ….
….) terms and utilizing equations (S3) and (S4), one can obtain,

$$\frac{k_z^2}{\frac{\left(\varepsilon_{x_2}d_2 + \varepsilon_{x_1}d_1\right)}{d}} + \frac{k_x^2}{\frac{d}{\left(\frac{d_1}{\varepsilon_{z1}} + \frac{d_2}{\varepsilon_{z2}}\right)}} = k_0^2 \tag{S19}$$

$$\frac{k_z^2}{\varepsilon_{x_{eff}}} + \frac{k_x^2}{\varepsilon_{z_{eff}}} = k_0^2 \tag{S20}$$

Now, defining filling fraction as,

$$f = \frac{d_1}{d} = \frac{d_1}{d_1 + d_2} \tag{S21}$$

From equations (S19), (S20) and (S21), one can obtain the effective permittivity values as,

$$\varepsilon_{x_{eff}} = \varepsilon_{x_1}f + \varepsilon_{x_2}(1-f) \tag{S22}$$

$$\varepsilon_{z_{eff}} = \left(\frac{f}{\varepsilon_{z1}} + \frac{1-f}{\varepsilon_{z2}}\right)^{-1} \tag{S23}$$

Effect of Top Layer (Interface Layer) on Transmission, Reflection and Absorption of GhHC

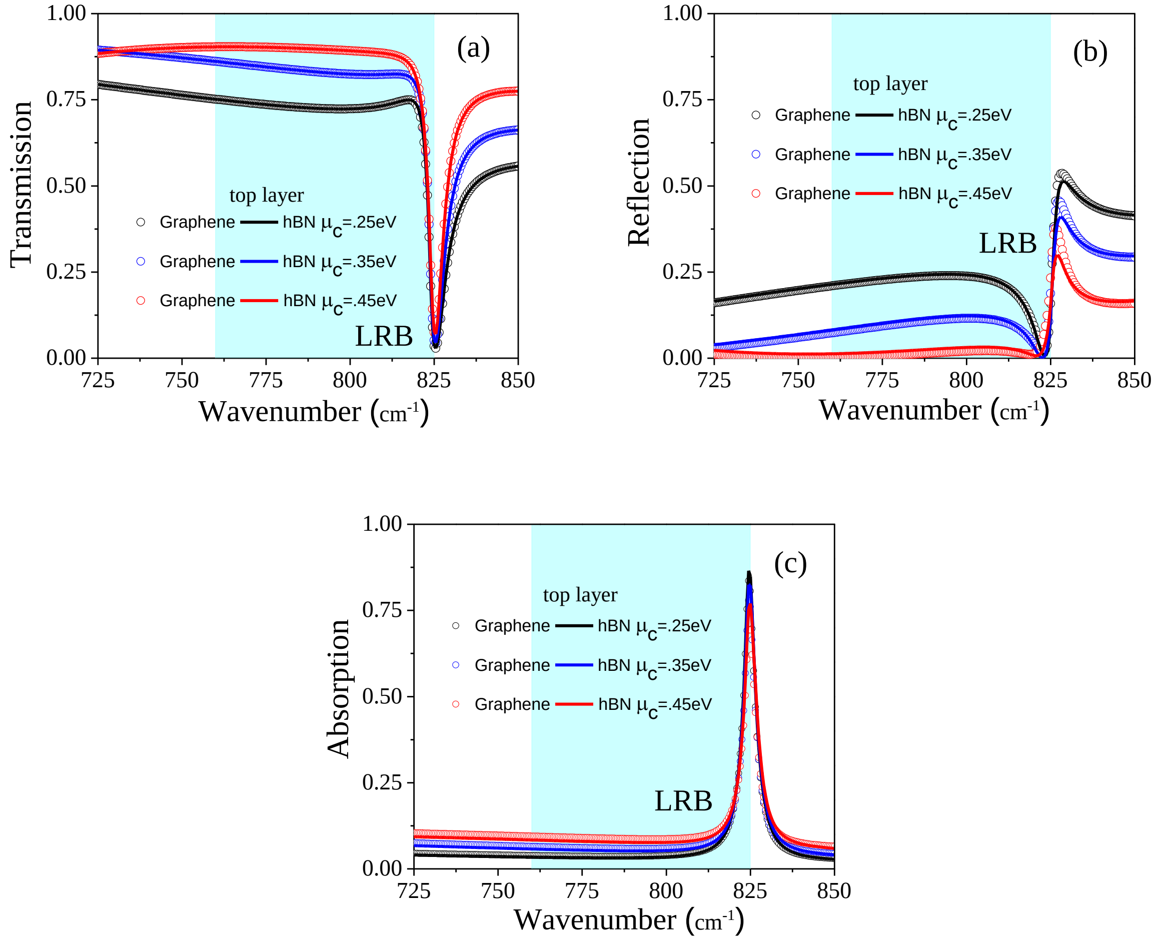

Figure S2. (a) Transmission (b) reflection and (c) absorption coefficient values of GhHC calculated by the transfer matrix method (TMM) as a function of wavenumber at incidence angle 30^0 and with different top layer. The solid lines represent the calculated values when the first layer of the GhHC is hBN and the symbols represent the calculated values when the first layer of the GhHC is graphene for different values of chemical potential of graphene (0.25eV, 0.35eV, 0.45eV). Total thickness of GhHC d=~1μm, thickness of graphene and hBN in the unit cell, d_1=1nm and d_2=100nm respectively, total number of unit cell N=10 and the relaxation time of graphene, τ=200fs. Shaded region represents the lower Reststrahlen band (LRB).

Figs. S2a, S2b and S2c show the transmission, reflection and absorption coefficients of GhHC as a function of wavenumber at incidence angle 30^0 for different values of chemical potential of graphene. Symbols represent the data when the first layer (top layer) of GhHC is graphene and solid lines represent the data when the first layer is hBN. Form Figs. S2a, S2b and S2c it can be clearly observed that in the LRB, top layer (first layer of GhHC) has very little effect on the transmission, reflection and absorption properties of GhHC.
